# Comparison of higher order aberrations in patients with various refractive errors

**DOI:** 10.12669/pjms.314.7538

**Published:** 2015

**Authors:** Muhammad Saim Khan, Sadia Humayun, Aisha Fawad, Mazhar Ishaq, Sabahat Arzoo, Fawad Mashhadi

**Affiliations:** 1 Dr. Muhammad Saim Khan, Armed Forces Institute of Ophthalmology, Rawalpindi, Pakistan; 2 Dr. Sadia Humayun, Armed Forces Institute of Ophthalmology, Rawalpindi, Pakistan; 3 Dr. Aisha Fawad, Armed Forces Institute of Ophthalmology, Rawalpindi, Pakistan; 4 Prof. Dr. Mazhar Ishaq, Armed Forces Institute of Ophthalmology, Rawalpindi, Pakistan; 5 Miss. Sabahat Arzoo, Armed Forces Institute of Ophthalmology, Rawalpindi, Pakistan; 6 Dr. Fawad Mashhadi, Armed Forces Institute of Ophthalmology, Rawalpindi, Pakistan

**Keywords:** Refractive Error, Visual acuity, Higher Order Aberration, Aberrometery

## Abstract

**Objective:**

To compare the mean root mean square (RMS) of total higher order aberrations (HOAs), coma and spherical aberrations in individuals with myopia, hypermetropia and myopic astigmatism.

**Methods:**

This prospective analytical study was conducted at Armed Forces Institute of Ophthalmology, Rawalpindi, Pakistan from Jan 2014 to Dec 2014. Two hundred eyes of 121 patients with age ranging from 18-40 years were included in the study. Patients were divided into 4 group namely Low myopia, High myopia, Astigmatism and Hypermetropia on the basis of refractive error. Included were the patients who had refractive error more than ± 0.5D and best corrected visual acuity (BCVA) of 0.00 or better. Patients who had history of surgery and / or eye disease were excluded from the study. Visual acuity (VA), Spherical equivalent (SE) of refractive error, RMS value of total HOAs, coma and spherical aberrations were evaluated. HOAs were measured with aberrometer (Wavelight analyzer version 1073) at 6 mm pupil size.

**Results:**

Age of the patients ranged from 18 years to 40 years with mean age of 29.10±10.6 years. Seventy one (35.5%) were males and 129 (64.5%) were female. Mean RMS value of HOAs, coma and spherical aberrations was calculated in all four groups. RMS of total HOAs and spherical aberrations in hypermetropia was 0.96±0.96 and 0.30±0.42 respectively and it was higher than other three groups.

**Conclusions:**

In overall comparison the mean RMS of total HOAs and spherical aberrations was significantly increased in hypermetropia group and there was a statistically significant negative correlation of SE of hypermetropia with RMS of total HOAs and spherical aberration.

## INTRODUCTION

Higher order aberrations (spherical aberrations, coma, trefoil) are small optical irregularities of the ocular refractive media. Unlike low order aberrations (myopia, hypermetropia, simple astigmatism) they cannot be corrected with spectacles or contact lenses. After successful correction of refractive error by refractive surgery, patients at times complain of halos, glare and decreased contrast. Authors have concluded that HOAs are responsible for these postoperative symptoms.^1^ Many studies in the past showed significant variability of HOAs among the individuals of same population. Popularization of laser corneal refractive surgery and its potential to induce or remove optical aberrations (both low order and higher order aberrations) is the main reason behind better understanding of the nature of aberrations as well as their influence on visual quality.^2^ In the normal eye, cornea is responsible for 90% of total aberrations and Zernike polynomials are most commonly used to describe these HOAs. Advent of new diagnostic modalities and introduction of wave front technology has brought revolution in the field of corneal refractive surgery. These advancements has expanded the indications of corneal refractive surgery beyond correcting only low order aberrations.^3^

Zernike polynomials are divided into several orders, low-order aberrations (first and second order), and high order aberrartions (third order onwards). Important higher order aberrations include coma, trefoil and spherical aberrations and they are measured by root mean square (RMS) value which represents the ocular aberrations in micrometers. Values of measured ocular aberrations in the Zernike polynomials are dependent on pupil diameter at the time of examination.^2^-^4^ HOAs and their relation to amount and type of refractive errors has been studied in a number of studies but the result are controversial. Some studies have shown no statistically significant correlation between HOAs and amount or type of refractive error,^5^-^7^ while others concluded a strong correlation of HOAs with myopia.^8^-^11^ Spherical aberrations were found significantly correlated with myopia by some authors,^8^ while others confined their relation to high myopia only,^12^ and still others could not found any statistically significant correlation.^11^

The rationale of conducting this study was to collect a local database of HOAs among various refractive errors in patients investigated for refractive surgery. The comparison of HAOs (in micrometers) with the amount and type of refractive error (in diopters) will help in management of these patients.

## METHODS

This prospective analytical study was conducted at Armed Forces Institute of Ophthalmology, Rawalpindi, Pakistan from Jan 2014 to Dec 2014. Sample size was calculated on the basis of WHO calculator and appeared to be 200 eyes. So a total of 200 eyes of 121 subjects were included in the study by non-probability (consecutive) sampling technique and two eyes of the same patient were considered independently. Most of the patients were evaluated to undergo refractive surgery. Included were the patients with age ranging from 18 – 40 years and refractive error more than ± 0.5 D, while those with history of ocular disease and amblyopia were excluded. We categorized the patients into four groups on the basis of mean spherical equivalent (Table-I).

**Table-I:** Groups of patients.

Groups	n (%)	Spherical equivalent	Mean Age	P value
Low Myopia	90 (45%)	-3.4 ± 1.5	23.5 ± 3.5	P < 0.05
High Myopia	30 (15%)	-7.0 ± 1.38	24.5 ± 3.0	P < 0.05
Astigmatism	38 (19%)	-2.9 ±1.20	23.5 ± 3.0	P < 0.05
Hypermetropia	42 (21%)	+2.0 ± 1.16	32.3 ± 8.33	P < 0.05

In order to generate more accurate and reliable results, patients were instructed to stop using contact lenses for at least 02 weeks prior to aberrometry. All patients were examined for Uncorrected visual acuity (UCVA) and corrected distant visual acuity (CDVA) followed by slit lamp examination to rule out conditions like dry eyes, corneal ectasia, corneal scar, cataract, media opacities, previous surgery or trauma. HOAs were measured by aberrometer (Wavelight Allergo Analyzer Version 1073) after instillation of cyclopentoalate (0.5%, 1 drop repeated after 15 mins) to paralyze the accommodation and dilate the pupil. Measurements were repeated three times for each eye and the best image was selected. Analysis was based on a pupil size of 6 mm. RMS value was calculated from Zernike coefficients and we calculated mean RMS of total HOAs, spherical aberrations and coma.

Statistical package for Social sciences (SPSS 17.0) for windows was used for statistical analysis. The data was described in terms of mean±SD (Standard deviation) for each group on the basis of type of refractive error. Linear regression analysis was used to assess the distributions of HOAs (total HOAs, coma, spherical aberration)and their correlation with amount and type of refractive error.(p<0.05 significance level)

## RESULTS

**Fig. 1:**
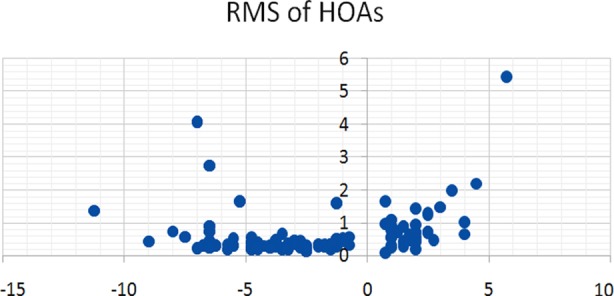
Total HOA and SE.

A total of 200 eyes of 121 patients were studied. Seventy one (35.5%) were males and one hundred and twenty nine (64.5%) were females. Age of the subjects ranged from 20 to 40 years with mean age of 29.1±10.6 years. Mean age of hypermetropes was 32.3±8.33 while other three groups were 23.5±3.5, 24.5±3.0, 23.5±3.0 for low myopia, high myopia and astigmatism respectively. Refractive error was described in terms of spherical equivalent and it ranged from +6.00 to - 10.50 D. RMS of total HAOs, spherical aberrations and coma were calculated and given in Table-II. The distribution of various HOAs across the range of refractive error is given in scatter graphs. (Fig. 1, 2 & 3). Correlation of SE of refractive error and various HOAs was analyzed and statistically significant is marked as * in Table-III.

**Table-II:** Mean RMS of total HOAs, spherical aberrations and coma in different groups.

	Mean RMS value		
	Total HOA	Coma	Spherical Aberration
Low Myopia	0.40 ± 0.31	0.21 ± 0.15	0.11 ± 0.07
High Myopia	0.92 ± 1.08	0.33 ± 0.24	0.11 ± 0.10
Astigmatism	0.36 ± 0.12	0.28 ± 0.12	0.13 ± 0.09
Hypermetropia	0.96 ± 0.96	0.38 ± 0.27	0.30 ± 0.42

**Fig. 2:**
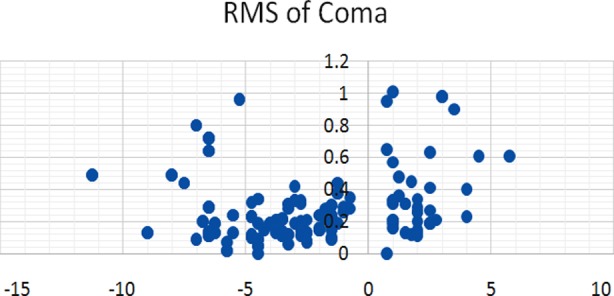
Coma and SE.

**Fig. 3:**
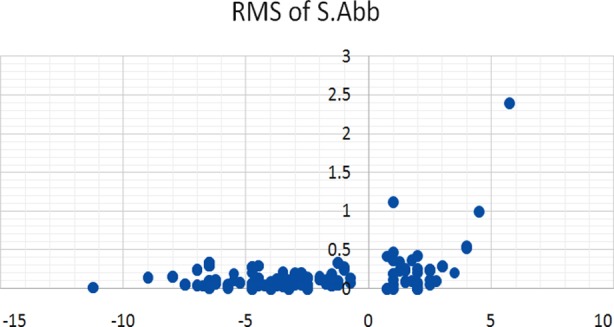
Spherical Aberration and SE.

**Table-III:** Shows correlation ‘r’ between SE of refractive error and HOAs in various groups.* indicates statistically significant results.

Refractive Error	Total HOAs	Coma	Sph. Abb
Low Myopia	-.017	-.090	-.278
High Myopia	-.026	.029	-.236
Astigmatism	-.025	.104	-.005
Hypermetropia	-.664*	.083	-.590*

## DISCUSSION

Aberrometry has proven to be invaluable in detecting eyes with an abnormal optical conditions especially in the context of corneal refractive surgery. Wavefront aberrations differed widely among the subjects, with a mean SD of approximately 0.10 um for RMS of Total HOAs. The summary of optical quality can be deduced from aberration coefficient and described as RMS value in micrometers. In our study, we investigated ocular HOAs in patients with myopia, hyperopia and astigmatism. The main focus was on coma (third order aberrations) and spherical aberrations (forth order aberrations), as these were the most significant components of HOAs.^13^-^16^ Authors have concluded the mean RMS of Total HOAs to be 0.33um for a 6.0-mm pupil.^5^,^17^-^20^

In our study, we found that HOAs were lower in myopes when compared to hypermetropes (Table-II). However this can be due to significantly higher mean age in hypermetropic group than myopes (Table-I) and studies have shown that HOAs increase with age.^5^,^17^-^19^ Marcos *et al*,^10^ reported that RMS of wavefront aberrations in high myopia (>6.0 D) has statistically significant positive correlation with refractive error. We in our study found out that there was no significant correlation of HOAs with amount of refractive error in all the groups except in hypermetropes. We, like He J *et al*.^9^ also found out no significant relation of myopic error with HOAs in contrary to Marcos *et al*. who conclude a positive correlation between the two (Table-III). Patients in hypermetropia group had statistically significant higher amount of spherical aberrations than other three groups as reported by Lorente *et al*.^20^ and Bisneto *et al*.^21^ We also found out a statistically significant negative correlation `r` of Hypermetropia with RMS of Total HOAs and Spherical aberration (marked with * in Table-III).

Some authors have concluded HOAs to be more in ametropia than emmetropic eyes^9^ while others proposed no relationship or even an opposite relationship between refractive error and HOAs^8^,^12^,^22^ and still others reporting greater higher order aberrations in myopes compared to hyperopes.^23^ Amount HOAs in our study were comparable to Chinese population however, we found our results to be higher when compared to Caucasians.^6^,^24^,^25^ The currently available literature regarding the clinical importance of HOAs and the potential benefit of correcting them made us study HOAs in our population. We believe that our results are important but studies on larger sample size are required to further evaluate, analyze and compare not only the changes in HOAs with refractive error but also their effect on patients` quality of vision.

## CONCLUSIONS

In overall comparison we noticed the mean RMS of total HOAs and spherical aberrations were significantly higher in hypermetropia group and there was a statistically significant negative correlation between SE of hypermetropia and RMS of Total HOAs.
